# NAFLD and Infection, a Nuanced Relationship

**DOI:** 10.1155/2021/5556354

**Published:** 2021-04-15

**Authors:** Abimbola Adenote, Igor Dumic, Cristian Madrid, Christopher Barusya, Charles W. Nordstrom, Libardo Rueda Prada

**Affiliations:** ^1^Division of Hospital Internal Medicine, Mayo Clinic Health System, Eau Claire, WI, USA; ^2^Mayo Clinic College of Medicine and Science, Rochester, MN, USA

## Abstract

The prevalence of nonalcoholic fatty liver disease (NAFLD) has increased significantly over the last few decades mirroring the increase in obesity and type II diabetes mellitus. NAFLD has become one of the most common indications for liver transplantation. The deleterious effects of NAFLD are not isolated to the liver only, for it has been recognized as a systemic disease affecting multiple organs through protracted low-grade inflammation mediated by the metabolic activity of excessive fat tissue. Extrahepatic manifestations of NAFLD such as cardiovascular disease, polycystic ovarian syndrome, chronic kidney disease, and hypothyroidism have been well described in the literature. In recent years, it has become evident that patients suffering from NAFLD might be at higher risk of developing various infections. The proposed mechanism for this association includes links through hyperglycemia, insulin resistance, alterations in innate immunity, obesity, and vitamin D deficiency. Additionally, a risk independent of these factors mediated by alterations in gut microbiota might contribute to a higher burden of infections in these individuals. In this narrative review, we synthetize current knowledge on several infections including urinary tract infection, pneumonia, *Helicobacter pylori*, coronavirus disease 2019, and *Clostridioides difficile* as they relate to NAFLD. Additionally, we explore NAFLD's association with hidradenitis suppurativa.

## 1. Introduction

Nonalcoholic fatty liver disease (NAFLD) is the most common chronic liver disease in the United States and worldwide. Its prevalence in the USA is about 24%, while it is around 30% in the Middle East and South America [1–3].

The hallmark feature of NAFLD is the aberrant and excessive storage of macrovesicular fat (in >5% of hepatocytes) due to alterations in the homeostatic balance between the fat synthesis and its utilization [1–3]. In some individuals, this seemingly benign fat accumulation in hepatocytes triggers inflammation leading to nonalcoholic steatohepatitis (NASH), liver fibrosis, cirrhosis, and/or development of hepatocellular carcinoma. The progression from NAFLD to these more severe entities is multifactorial and depends on an individual's genetic factors, environmental factors, and abnormal activation of the innate immune system [1–5]. Abnormal activation of the innate immune system leads to persistent low-grade inflammation which leads to tissue injury and fibrosis and has an important role in carcinogenesis [1–5].

The liver, in addition to being a vital metabolic organ, also plays a significant role in the human immune system. Liver macrophages (Kupffer cells) and lymphocytes constitute about 20% of total liver cells [6], and they are the first immune cells to process various antigens and pathogens from the gastrointestinal tract. While the role of the immune system is well recognized in the pathogenesis of NAFLD and its complications, it is less known how the presence of NAFLD influences an individual's risk for the development of various bacterial, fungal, and viral infections.

Individuals with NAFLD commonly have one or more elements of metabolic syndrome including obesity, insulin resistance, dyslipidemia, and systemic hypertension. Obesity and type II DM (T2DM) have been previously recognized as risk factors for the development of various infections [7–10]. While patients with NAFLD might have a higher risk for infections due to the concomitant presence of obesity and/or T2DM, some studies have demonstrated an increased risk for bacterial infections independent of the presence of metabolic syndrome in this population [11].

The exact mechanism by which patients with NAFLD might be more prone to the development of infection remains unclear; however, there are several theories. Steatosis might derange sinusoid microcirculation leading to impaired hepatic microbial clearance. Decreased vitamin D levels in NAFLD likely impair innate immunity [12, 13]. There is evidence that NAFLD patients have impaired function of hepatic natural killer cells [14–16]. The impaired function of the Kupffer cells and their aberrant activation might contribute not only to the development of NAFLD but also to an increased risk of infection [17, 18]. Additionally, impaired function of neutrophils in the setting of insulin resistance [19], a higher incidence of small bowel intestinal overgrowth, and dysfunction of the tight junctions of small bowel epithelium causing increased intestinal permeability [20,21] could all further contribute to infection risk in this patient population.

When analyzing the relationship between liver disease and infection, it is important to attend to the nature of the said infection. Untreated infections with hepatotropic viruses such as hepatitis B and hepatitis C (in conjunction with host immune response) cause liver inflammation, fibrosis, and eventually cirrhosis. For these patients, the infection and immunological response of the host are the primary events in the pathogenesis of liver cirrhosis. Conversely, other infections (i.e., fungal and bacterial) are rather the *consequence* of liver cirrhosis and appear dependent on the severity of the disease. It is paramount to recognize that patients with liver cirrhosis have 4-fold higher mortality than patients without cirrhosis [22] and those who develop sepsis have a staggering mortality of up to 75% [23]. Hence, the development of bacterial infection and/or sepsis in the cirrhotic patient has been recognized as a distinct stage in the natural progression of the liver disease [24–26]. Furthermore, bacterial infections are a well-recognized trigger for acute on chronic liver failure, which is also associated with increased mortality [26].

A partial SIRS-like state coupled with negative cultures in 30–50% of infections can make it difficult to differentiate infected from uninfected patients in liver cirrhosis [27]. Cirrhotic patients are also in a state of immune dysfunction combined with a state of excessive activation of proinflammatory cytokines which has been described as “cirrhosis-associated immune dysfunction syndrome” [28]. Immune dysfunction is explained by a decrease in phagocytic activity and a reduction in serum albumin, complement, and protein C activity, along with impaired opsonic activity in serum and ascitic fluid [28, 29]. Additionally, an excessive response of proinflammatory cytokines predisposes to the development of serious complications such as shock, liver failure, renal failure, and death once infection occurs.

The risk of infection appears commensurate with the progression of liver disease from steatosis through cirrhosis. Infection risk likely corresponds to several mechanisms related to the development of portal hypertension, bowel edema, and ascites. Bacterial translocation is the major pathogenetic factor contributing to infections in liver cirrhosis. Alterations to the gut microbiome, increased acid suppression, and increased intestinal permeability in cirrhosis contribute to bacterial overgrowth and enhanced bacterial translocation from the gut to the systemic circulation and ascitic fluid [30]. With the progression of fibrosis in NAFLD, gut dysbiosis has been identified (via gut microbiome-based metagenomic signature) to cultivate more Gram-negative organisms [31]. This is relevant because Gram-negative bacteria are more commonly implicated in bacterial infections with chronic liver disease. Alterations to the microbiome in conjunction with the increased risk of bacterial translocation [20, 21] demonstrate a correlation between the risk of infection and the severity of liver disease.

Additionally, vitamin D deficiency likely has negative impacts on innate immunity [12, 13], thereby contributing to an increased risk of bacterial infections. An inverse relationship has been identified between vitamin D levels and severity of NAFLD [32], which further implicates the progression of liver disease with the risk of infection.

Lastly, a study by Nseir et al. reviewed the association of NAFLD with 30-day all-cause mortality in adult patients admitted with community-acquired pneumonia [33]. The study found that the association was stronger in those with advanced fibrosis (fibrosis score >2) compared to early fibrosis (fibrosis score 0–2), suggesting that disease outcome is correlated with the severity of fibrosis.

The aim of this narrative review is to synthetize data on infection complications in patients with NAFLD, to raise awareness regarding the potential association between these entities, and to promote further research in this area.

## 2. Methodology

We have used PubMed/Medline database using the following keywords: “NAFLD and infection,” “NAFLD and cellulitis,” “NAFLD and *Clostridioides difficile* or/and *Clostridium difficile*,” “NAFLD and pneumonia,” and “NAFLD and COVID 19” to select studies and review articles related to this topic. Articles describing alteration of the immune system as it relates to the pathogenesis of NAFLD were reviewed to a lesser extent as it is not the main goal of this review article. Due to the overall insufficient number of studies (retrospective or prospective) on the risk of infection in people with NAFLD, we were unable to use PRISMA guidelines for a systematic review of literature; rather, we have included these studies in this narrative review.

Due to the recent change in nomenclature, both terms Clostridium and Clostridioides were used in the literature search. A manual review of the references from the articles identified through the database literature search was done to increase the comprehensiveness of the literature review. Articles in languages other than English and Spanish were excluded.

### 2.1. NAFLD and Bacterial Pneumonia

Pneumonia is a major cause of morbidity and mortality, particularly at the extremes of ages. In the United States, 43,881 (13.4/100 000 population) people passed away from pneumonia in 2019 [34].

A retrospective study by Nseir et al. demonstrated the association between NAFLD and recurrent bacterial infections which appears to be independent of metabolic syndrome [11]. In that study, the incidence of bacterial respiratory tract infections was second only to those involving the urinary tract. Another retrospective review of 141 patients admitted with pneumonia further corroborated the association between pneumonia and NAFLD. In the study, 40.4% of the study group showed evidence of NAFLD compared to 27.6% of patients in the control group [35]. Posttraumatic ventilator-associated pneumonia also appears to be associated with NAFLD. Bailey and Parikh [36] demonstrated an increased prevalence of posttraumatic ventilator-associated pneumonia in NAFLD patients admitted to the critical care unit when compared to patients with other similar risk factors, but without NAFLD. Another retrospective study by Nseir et al. [33] showed an increased mortality in patients with community-acquired pneumonia with NAFLD compared to those without (17% vs. 5.82%). This finding suggests that NAFLD may be related to all-cause mortality in patients with community-acquired pneumonia, an association that became more obvious in those with more severe hepatic fibrosis.

The pathophysiologic mechanisms of pneumonia as they relate to NAFLD have not been clarified in any of the studies. Again, the association of NAFLD with T2DM and obesity is a potential link. Suboptimal functioning of neutrophils in terms of adherence, chemotaxis, phagocytosis, and bactericidal activity is seen in diabetic patients, predisposing them to infections [37], more so with coexisting acidosis [38]. The antioxidant pathways of neutrophils of diabetic patients (e.g., superoxide dismutase) also appear to malfunction [39]. Falguera et al. showed that diabetic patients are more likely to have severe pneumonia with associated pleural effusions as well as higher mortality [40]. Alterations in neutrophil functions described above likely contribute to the predisposition to pneumonia. Although some studies noted no difference in mortality from pneumonia among obese and nonobese patients [41, 42], obese patients are more likely to develop both pneumonia and more severe pneumonia [43]. Adipose tissue is involved in the generation of inflammatory mediators, e.g., leptin and adiponectin [44]. While adiponectin suppresses immunity, leptin is involved in the activation of the immune system. Leptin resistance therefore could potentially be one of the links between obesity and infection. Another mechanism is the participation of adipose tissue in chronic low-grade inflammation [44]. Patients with lean NAFLD [45, 46] are still predisposed to bacterial infections; however, no previous study has compared the risk of bacterial infections between lean and nonlean NAFLD. Given the recent studies that suggest an association of NAFLD with recurrent bacterial infections, there is likely an association which is independent of metabolic syndrome elements. Most likely the association is related to variations in immunity.

### 2.2. NAFLD and COVID-19

In COVID-19 infections, NAFLD has been found to be a more significant risk factor for hospital admission when compared to age, gender, obesity, or other comorbidities. NAFLD also appears to account for the risk attributed to obesity in COVID-19 which highlights the importance to screen patients with elevated BMI for NAFLD [47].

All human coronaviruses can cause liver injury. Angiotensin-converting enzyme 2 (ACE2) present in liver and biliary epithelial cells acts as the cellular entry receptor for SARS-CoV-2, making the liver susceptible to infection [48]. The cellular transmembrane serine protease 2 (TMPRSS2) is also a critical factor to enable cellular infection by coronaviruses [49]. TMPRSS2 cleaves the SARS-CoV-2 spike protein which allows fusion of the viral and cellular membranes. Systemic inflammation and adverse drug reactions are the main mechanisms of liver injury in severe coronavirus disease. ALT/AST elevation and acute liver injury have been found in 23% and 2% of COVID-19 patients, respectively. Regular monitoring of liver function during hospitalization is important. Both ALT/AST elevation and acute liver injury have been found to be independently associated with adverse clinical outcomes such as ICU admission, mechanical ventilation, and death in COVID-19 patients [50]. When present, it is difficult to differentiate whether the elevation of liver enzymes is due to COVID-19 infection, complications of the disease, or secondary to drug-induced liver injury (DILI).

Several studies suggested that obesity, hypertension, and diabetes greatly increase the risk of severe and prolonged COVID-19 infection [51–53]. As these conditions are commonly present in NAFLD, it is intuitive to see the association between NAFLD and severe COVID-19 disease. It has been postulated that the chronic proinflammatory state associated with these metabolic diseases may play a role, at least in part due to an activation of the renin-angiotensin system [54–56]. This preexisting proinflammatory state seems to favor the cytokine storm, which may result in the multiorgan failure observed in severe COVID-19 [57–59].

The hepatic expression of ACE2 and TMPRSS2 remains unchanged in patients with NAFLD but is downregulated in women, indicating a protective role of estrogens in liver injury caused by SARS-CoV-2 [60–62]. Obese patients are differently affected by T2DM and NAFLD. Obese women with diabetes have unexpectedly lower levels of ACE2 and TMPRSS2 compared to obese normoglycemic women. Conversely, obese patients with NASH show markedly higher expression of these genes, suggesting that advanced stages of NAFLD might predispose individuals to COVID-19 [61].

A study evaluated 202 consecutive patients with confirmed COVID-19 and NAFLD status based on liver ultrasonography. Liver injury was observed in 101 (50%) and 152 (75.2%) patients on admission and during hospitalization, respectively. Almost all liver injury was mild with a hepatocellular pattern (elevation in ALT). Compared with non-NAFLD subjects, patients with NAFLD had a higher risk of disease progression to severe COVID-19 and longer viral shedding time [63].

Some investigational treatments of COVID-19 have shown uncertain clinical benefit, and their use may be controversial. These consist of antivirals, antibiotics, antifungals, monoclonal antibodies, immune-modulatory agents, anticoagulants, and sedative agents which may be hepatotoxic and cause drug-induced liver injury (DILI). The said treatments may aggravate preexisting liver lesions classically observed in NAFLD including fatty liver, necroinflammation, and fibrosis or trigger the transition of simple fatty liver to NASH [64]. DILI in COVID-19 patients may be worsened by acute heart failure and/or acute kidney injury due to the altered pharmacokinetics of these medications. Hospitalized COVID-19 patients are often polymedicated and at risk for multiple drug-drug interactions and drug-induced adverse events.

While liver injury is not the primary cause of death in COVID-19 patients, hepatic dysfunction can worsen the overall patient's condition, and patients with NAFLD seem to be particularly more vulnerable to these complications. Therapies directed to the treatment of metabolic disease may mitigate the risks from NAFLD.

### 2.3. NAFLD and *Helicobacter pylori*

*Helicobacter pylori* is a Gram-negative microaerophilic bacterium that commonly colonizes the stomach in humans. Its prevalence in developed nations is 20% whereas in developing nations its prevalence may be as high as 70%. *H. pylori* is the most important risk factor for chronic gastritis, peptic ulcers, gastric cancer, and gastric mucosa-associated lymphoid tissue (MALT) lymphoma. Growing evidence has linked *H. pylori* to many extra gastrointestinal ailments including obesity, T2DM, ischemic heart disease, and idiopathic thrombocytopenic purpura [65]. There has been a particularly intense focus on the association between *H. pylori* and NAFLD following a 2008 study by Cindoruk et al. which found *H. pylori* 16S rDNA in a liver biopsy of a patient that had NASH. Subsequent research studies have sought to identify any association between *H. pylori* and liver disease in hope of finding an actionable treatment for this growing problem [66]. Some studies have tried to show an association between *H. pylori* eradication and liver fat content and its function. Other studies have tried to investigate the possible function of *H. pylori* in NAFLD pathogenesis particularly as mediated by insulin resistance [67].

While some studies have illustrated a possible association between *H. pylori* and NAFLD, the preponderance of evidence currently available does not support this.

### 2.4. NAFLD and Hidradenitis Suppurativa

Hidradenitis suppurativa (HS) has been related to NAFLD [68]. At present, there are limited studies looking at the association of HS and NAFLD, but there can be a synergism between bacterial infection and HS leading to this condition.

Hidradenitis suppurativa (HS), also known as acne inversa or Verneuil's disease, is an inflammatory condition of the skin that affects the hair follicle. It usually presents after puberty with painful lesions in the apocrine gland-bearing areas of the body. The most common areas include axillae, inguinal, and anogenital regions [69]. The exact etiology of HS is unknown, but associated factors to developing HS include genetics [70], mechanical stress to the skin [71], smoking [71], hormones [72], obesity [73], and bacteria [74, 75].

It is also considered a systemic inflammatory disease of the terminal follicular epithelium of the apocrine glands, with a prevalence of 0.05% to 4.10%. Interestingly, one cohort study found the prevalence of NAFLD in patients with hidradenitis to be 38.5% [76].

HS was found to be an independent factor for the development of NAFLD after adjusting for classic cardiovascular and steatosis risk factors (OR, 7.75; 95% CI, 2.54–23.64; *P* < 0.001) [68]. In another study, a total of 125 patients with hidradenitis and 120 patients without it were recruited, matched for age, sex, and body mass index (<25 or ≥25 kg/m^2^). Both groups presented similar proportions of overweight or obesity (89.6% vs. 90%). Patients with HS had a significantly higher prevalence of NAFLD compared with those who did not (57.6% vs. 31.7%, *P* < 0.001). Multivariable analysis confirmed an independent association between HS and NAFLD (odds ratio, 2.79; 95% confidence interval, 1.48–5.25; *P* = 0.001) [77].

Both NAFLD and HS are associated with several other conditions including obesity, dyslipidemia, and insulin resistance [78–80]. After identifying HS as an independent risk factor, it has been hypothesized that the possible explanation for developing NAFLD may be related to the chronic inflammation due to persistent and abnormal secretion of adipokines and several proinflammatory cytokines [81, 82]. Bacterial infection seems to have a role as a synergistic factor in chronic inflammation as postulated by Nikolakis et al. in a review of prospective studies and one retrospective study. While HS is not considered a primary infectious disease, HS is thought to be a skin condition that predisposes to infection, thereafter causing chronic inflammation [75]. The efficacy of targeted antibiotic therapy favors and supports this hypothesis [83–85]. Interestingly, one case-control study found that the microbiome in HS versus non-HS patients is significantly different, and suggests a link between dysbiosis and HS [86].

The proposed mechanism by which HS leads to NAFLD includes the upregulation of proinflammatory cytokines (IL-1b, IL17, and TNF-alpha) [87]. There is also a significant expression of IL-12/Th1 and IL-23/Th17 [88]. Similar to psoriasis, another inflammatory skin condition strongly associated with NAFLD [89–91], the pathogenesis is felt to overlap in the following way: tumor necrosis factor-alpha and interleukins 1, 2, 6, and 17 influence glucose metabolism and insulin sensitivity in hepatocytes and adipocytes causing uncontrolled lipolysis and increased hepatic free fatty acid deposition [78, 92]. Adiponectin, another anti-inflammatory hormone associated with glucose metabolism and insulin sensitivity, is also decreased in patients with HS [81]. Interestingly, to support this pathogenesis, liver biopsies in a patient with NAFLD have revealed hepatic distribution RNA of the inflammatory cytokines TNF-alpha and the adiponectin with its receptors [47, 93].

### 2.5. NAFLD and Urinary Tract Infections

Urinary tract infections (UTIs) are rampant in all patient populations, both in the community and in hospitals. A population-based review of laboratory data for residents of the Calgary health region in 2004/2005 showed an annual incidence of 17.5 per 1000 people [94]. Despite the fact that numerous bacteria invade the urinary tract, it is an interaction between individual host factors and the virulence of the organism that eventually determines whether a UTI ensues or not [95].

Established risk factors for UTI include malformations of the urinary tract, female sex, genetic predisposition, and sexual activity [95]. NAFLD has also been identified as a risk factor for bacterial infection [11]. The most obvious pathophysiologic explanation of this is through NAFLD's association with metabolic syndrome.

Previous studies have showed an association between obesity and UTI [96–99]. The association was felt to be more significant in males [96–98] likely due to the positive influence of abdominal obesity on prostatic volume [97]. The association between obesity and urinary tract infection has also been shown to be independent of the association between T2DM and vitamin D deficiency. In premenopausal, nonpregnant women, obesity predisposes not only for a UTI but for recurrent episodes as well [99].

T2DM has long been shown to predispose patients to a number of infections, including those involving the urinary tract [100–102]. Hyperglycemia, diabetic nephropathy, neurogenic bladder, and a malfunctioning innate immune system are all perceived to be contributory [100].

More recent studies are pointing to the fact that NAFLD may be related to urinary tract infections by pathophysiologic mechanisms distinct from those associated with metabolic syndrome. Nseir et al. recently completed a retrospective case-control review of recurrent UTI in premenopausal women admitted to the hospital [103]. In this study, the incidence of NAFLD was higher in the group of patients with recurrent UTI than in the controls (43.5% vs. 21.5%), raising the probability of an association between the two entities. It also showed that patients with recurrent urinary tract infection were more likely to be vitamin D-deficient.

There also seems to be an independent association between NAFLD and vitamin D deficiency [32,104,105], with the degree of deficiency related to the severity of nonalcoholic fatty liver disease [32]. Vitamin D deficiency may independently increase the risk of UTI given that vitamin D is known to stimulate the cathelicidin, an antimicrobial peptide that can be found in the epithelial cells of the urinary bladder [97, 106].

Multiple studies and even meta-analyses have shown an association between nonalcoholic fatty liver disease and urolithiasis [107–109], potentially illustrating another pathophysiologic mechanism for urinary tract infections in this chronic illness. Finally, contributions from defects in both innate and adaptive immunity cannot be ruled out.

More studies (and especially prospective studies) are needed to confirm and further investigate the strength of the association between urinary tract infection and NAFLD. This will potentially open up new avenues for the prevention and management of urinary tract infections in this population of patients [110].

### 2.6. NAFLD and *Clostridioides difficile*

While firm evidence is still lacking, it has been postulated that patients with NAFLD might have an increased risk for the development of *Clostridioides difficile* colitis (CDC). Additionally, it has also been postulated that CDC can trigger changes associated with the development of NAFLD [111].

Papic et al. identified NAFLD as an independent predictor for CDC development [111]. In their study, they followed 314 patients of which 83 had NAFLD and 231 were controls, with the NAFLD group demonstrating higher rates of CDC development compared to the controls. Similarly, Bishara et al. [112] recognized that CDC was more frequently diagnosed in patients with higher body mass index (BMI), and they found obesity to be an independent risk factor for CDC development [112].

We postulate that changes in intestinal microbiota in both CDC and NAFLD might be the common denominator and linked with obesity. Since obesity is associated with changes in intestinal microbiota and is commonly found in patients with NAFLD, the association is relatively evident.

The development of CDC is related to derangements in the intestinal microbiota [113], and it has been demonstrated that *Bacteroides* and *Bifidobacterium* play an important role in the mechanism preventing colonization by *C*. *difficile* [114]. Studies have shown that concentrations of *Bacteroidetes* in the intestines of CDC patients are lower, while intensities of *Firmicutes* and *Proteobacteria* are higher compared to controls [115]. Bearing in mind that obesity has been associated with a relative decrease in the proportion of *Bacteroides* to *Firmicutes* [116], it is not surprising that obese patients might be more susceptible to CDC development.

Similarly, studies on intestinal microbiota in NAFLD patients demonstrated an increase of the phylum *Firmicutes*, *Lactobacillus*, and several genera within the Lachnospiraceae family compared to the healthy individuals [31]. Furthermore, it has been demonstrated that microbiome compositions differ in patients with mild or moderate NAFLD compared to people with advanced fibrosis. By using whole-genome shotgun sequencing of DNA extracted from stool samples, it has been shown that a higher prevalence of *Firmicutes* is present in people with mild or moderate NAFLD, while *Proteobacteria* were dominant microbiota in those with more advanced liver fibrosis [117]. One can draw the conclusion that the risk for CDC might be higher in patients with advanced fibrosis compared to individuals with less severe forms of NAFLD.

Gut microbiota is linked not only to the development of NAFLD but also to its progression. Altered gut microbiota composition and function, together with visceral adipose tissue (VAT) accumulation, results in an imbalance between pro- and anti-inflammatory cytokines. This is considered the main cause of protracted inflammation in NAFLD and potentially the subsequent increased risk for the development of various infections including CDC.

## 3. Conclusion

There is strong evidence in the literature that patients with decompensated cirrhosis are at high risk for infectious complications. In patients with NAFLD (but without advanced fibrosis or cirrhosis), however, the evidence is less firm. Studies on this topic are scarce, and of those that exist, many are limited by small sample size or design. The studies are either retrospective or small, single-institution prospective studies. The evidence to date does seem to support an increased risk of infection; however, the extent of this association remains unclear. For certain infections such as pneumonia or UTI, the risk seems to exist primarily through the shared pathophysiology with T2DM and obesity. Hyperglycemia and insulin resistance lead to dysfunctional neutrophils, changes in innate immunity, and possibly vitamin D deficiency. A few studies, however, have demonstrated an increased infection risk in NAFLD even in those without obesity or T2DM. It is possible that the persistent low-grade inflammation associated with the accumulation of fat tissue may change the microstructure of liver tissue and possibly impair the function of liver macrophages (Kupffer cells). In other infections such as CDC, it seems that changes in microbiota in those with NAFLD promote *Clostridioides difficile* colonization and the development of infection. With COVID-19, increased and deregulated cytokine activity has been the main denominator for increased mortality, and patients with NAFLD seem to be particularly vulnerable. Additionally, low-grade inflammation and cytokine derangements are responsible for inflammatory skin changes such as HS. Further research through more rigorous multicenter prospective studies is urgently needed to address the questions regarding this population's risk of infection. As the link is more firmly established, then strategies for treatment and prevention may become evident.

## Data Availability

All data are publicly available.
